# Effect of cobalt(II) chloride hexahydrate on some human cancer cell lines

**DOI:** 10.1186/s40064-016-2405-0

**Published:** 2016-06-30

**Authors:** Sonia Mahey, Rakesh Kumar, Rohit Arora, Jyoti Mahajan, Saroj Arora, Renu Bhardwaj, Ashwani Kumar Thukral

**Affiliations:** Department of Botanical and Environmental Sciences, Guru Nanak Dev University, Amritsar, Punjab 143005 India; Department of Botany, DAV University, Jalandhar, Punjab 144012 India

**Keywords:** Apoptosis, Antiproliferative, CoCl_2_·6H_2_O, MTT

## Abstract

The present study investigates the anti-proliferative and apoptosis inducing mechanism of CoCl_2_·6H_2_O in PC-3 cancer cell line. Preliminary, three different forms of cobalt i.e., cobaltous (CoCl_2_·6H_2_O), macro-Co(II,III) oxide and nano-Co(II,III) oxide were screened for antiproliferative activity in PC-3 cell line. The CoCl_2_·6H_2_O being the most effective antiproliferative agent, hence it was further tested against lung (A549), prostrate (PC-3) and brain (IMR-32) cell lines. Human embryonic kidney cell line (293T) was used as a normal cell line to compare the toxicity of CoCl_2_·6H_2_O. The CoCl_2_·6H_2_O induced morphological and anatomical changes in PC-3 cancer cell which were studied using light, confocal and scanning electron microscopy. The lactate dehydrogenase was estimated which showed mild necrotic mode of cell death. The Annexin/PI staining confirmed the apoptotic mode of cell death in PC-3 cells. Further, production of reaction of reactive oxygen species and changes in mitochondrial membrane potential was also assessed spectrofluorimetrically. The cell cycle arrest was also investigated using flow cytometery. Finally, the caspase activity was estimated in CoCl_2_·6H_2_O treated PC-3 cancer cell line. Interestingly, it was found that CoCl_2_·6H_2_O induces more cell death in cancerous cells as compared to normal non-cancerous cells. These findings presented CoCl_2_·6H_2_O as potential antiproliferative agent.

## Background

The d-block elements confined to 3–12 groups of periodic table represent transition metals. They along with their complexes have found use in drug development since last decade (Gianferrara et al. 2009; Li et al. 2015). Their ability to exist in a number of oxidation states and reacting with other oppositely charged species has been exploited in medicinal chemistry. Some of the transition metals have been reported to possess antimicrobial, antifungal, antidiabetic and anticancer properties (Singh 2014). Different metal complexes viz., Ru(III), Co(II), Cu(II) nitrate, Pd(II) chloride, and coordination complexes of Pt(II), As, Sb, Bi, V, Fe, Rh, Ti and Ga have been investigated for their anticancer activities by various workers (Olivova et al. 2011; Sharma et al. 2008; Soldevila-Barreda et al. 2015; Bandyopadhyay et al. 2014; Chitambar 2013; Geldmacher et al. 2012; Obeid et al. 2012; El-Boraey and El-Din 2014; Novotny and Kombian 2014; Mojic et al. 2014; Via et al. 2014; de Assis et al. 2016; Huang et al. 2016). It has been reported that cytotoxic drugs specifically target frequently dividing cells because of the increased synthesis of nucleic acids in them during cell division (Thind et al. 2008).

Cobalt (Co) is a transition metal which is required as a trace element in both plants and animals. Co exists in numerous inorganic complexes with different oxidation states, but not all the oxidation states are medicinally important. It is a water soluble crystalline complex which gets decomposed on electrolysis. It is used in coloration of paint and glass. Cobalt(III) oxide is an insoluble (Mahey and Thukral 2014) non-conductive complex having optic and ceramic properties. Co plays a major role in the form of cobalamin i.e., vitamin B-12 by maintaining neurological and immune responses, red blood cell formation, synthesis of DNA and in development as well as growth of infants (Andres et al. 2010; Krisch et al. 2013; Hunger et al. 2014; Rush and Yajnik 2014; Chellan and Sadler 2015). It has also been reported as a cardio-, reno- and neuro- protective agent (Salnikow et al. 2000). CoCl_2_·6H_2_O has been reported to be used as a doping agent by athletes for improving their performance (Lippi et al. 2005).

The present study was designed to screen the antiproliferative activities of three different forms of cobalt i.e., cobaltous (CoCl_2_·6H_2_O), macro-Co(II,III) oxide and nano-Co(II,III) oxide against PC-3 cancer cell line. The most active form of Cobalt was further tested against three cancer cell lines viz., A549 (lung), PC-3 (prostrate), IMR-32 (brain) along with 293T (human kidney embryonic cells) employing MTT assay. Further, the mechanistic studies were carried out on PC-3 cell line using CoCl_2_·6H_2_O. Various mechanistic studies gave an insight into process of cellular death, i.e., apoptosis or necrosis in PC-3 cells. Assays employed for this purpose involved microscopic (phase contrast, confocal, scanning electron microscopy) investigations, measurement of reactive oxygen species (ROS), mitochondrial membrane potential (MMP), sub G_0_ population using flow cytometry, lactate dehydrogenase (LDH), annexin V-FITC/PI and caspase-3 activity.

## Methods

### Chemicals and reagents

Dulbecco’s modified Eagle’s medium (DMEM), Roswell Park Memorial Institute medium (RPMI), foetal bovine serum (FBS), penicillin, streptomycin, 4′,6-diamidino-2-phenylindole (DAPI), rhodamine123 (RH-123), 5,6-chloromethyl-2′7′-dichlorodihydrofluorescein diacetate (CM-H2DCFDA) and 3-(4,5-dimethylthiazol-2-yl)-2,5-diphenyltetrazolium bromide (MTT) were purchased from Sigma-Aldrich Corporation (St. Louis, MO, USA). Gentamicin was purchased from, Abbott Healthcare Pvt. Ltd CoCl_2_·6H_2_O, Macro-Co(II,III) oxide and Nano-Co(II,III) oxide were procured from Sigma-Aldrich Corporation (St. Louis, MO, USA).

### Procurement of cell lines

Cancer cell lines A-549, IMR-32, PC-3 and normal human embryonic cell line 293T used in the present study were procured from National Centre for Cell Sciences (Pune, India). The cell lines were grown in culture flasks containing nutritive medium (DMEM or RPMI) supplemented with 10 % FBS at 37 °C in CO_2_ incubator (5 % CO_2_ level + 90 % relative humidity). The cells were trypsanized and seeded in well plates as per the requirement of experiment.

### Sample preparation

Different concentrations of CoCl_2_·6H_2_O, Macro-Co(II,III) oxide and Nano-Co(II,III) oxide (10, 25, 50, 75, 100 mg/L) were prepared for the evaluation of antiproliferative activity. The sample was dissolved in growth medium (DMEM and RPMI) in which the cancer cell line was grown. The treatments design of the different samples was divided into various groups as follows:

1. X1: Control group: comprises of medium only (DMEM/RPMI)
2. X2: comprises of medium + CoCl_2_·6H_2_O
3. X3: composed of medium + CoCl_2_·6H_2_O + cell line
4. X4: composed of medium + cell line

Absorbance of CoCl_2_·6H_2_O only was calculated by (X2–X1); % inhibition was calculated by using formula

$$\left\{ {{\text{X}}4 - \left[ {{\text{X}}3 - \left( {{\text{X}}2 - {\text{X}}1} \right)} \right]} \right\} \times 100 / {\text{X}}4$$

Although the cancer cells used in the experiment were from single stock, still before each experiment the cells synchronization was done prior to the treatment by serum starvation method.

### MTT [3-(4,5-dimethylthiasol-2-yl)-2,5-diphenyltetrazolium bromide] colorimetric assay

To assess the antiproliferative activity of the samples, MTT assay was performed (Heckenkamp et al. 1999). The cells were seeded in 96 well plate at density of 1 × 10^4^ cells/well. After 24 h, the cells were treated with different concentration of 0.1 mL sample (5–100 mg/L) for 24 h. After treatment, the 0.02 mL of MTT dye (2.5 mg/mL) was added to each well and incubated for 4 h. After this to each well is added 0.1 mL of DMSO to solubilised formazan crystals. These plates were then read on ELISA plate reader at 570 nm. All the treatments were made in triplicates. Camptothecin at a concentration of 10 µM was used as positive standard.

### Revival of treated PC-3 cell

The PC-3 and 293T cells (1 × 10^4^) in 96-well plate were treated with IC_50_ of CoCl_2_·6H_2_O for 24 h. The non-adhered cells which come up in the medium and usually regarded as dead cells were harvested by collecting the medium in sterilized 10 mL centrifuge tubes. The medium containing cells were centrifuge at 1500 rpm for 5 min. The medium was discarded and cell pellet was mixed with fresh nutritive medium. The cells along with nutritive medium were transferred in culture flask and incubated in CO_2_ incubator for 24 h. After incubation, the cells were observed under microscope if they survived the treatment of CoCl_2_·6H_2_O and were photographed.

### Microscopic studies of CoCl_2_·H_2_O treated PC-3 cells

The morphological and anatomical changes induced in PC-3 cells by CoCl_2_·6H_2_O were studied with the help of different microscopic techniques.

### Light microscopy

The light microscopy of treated PC-3 cells was done using the previously studied method with slight modifications (Ramasamy et al. 2013). The PC-3 cells were incubated in 6 well plate with density of 5 × 10^5^ cells/well. After 24 h, the cells were treated with different concentrations (5, 10, 25, 50, 75 and 100 mg/L) of CoCl_2_·6H_2_O for 12–14 h. After treatment, the cells were observed under phase contrast microscope and changes in the morphology of PC-3 cells were photographed.

### Confocal microscopy

The confocal microscopy was done using previously followed method with slight modification (Bhushan et al. 2007). The PC-3 cells (5 × 10^5^ cells/well) were seeded in 6-well plate and allowed to adhere for 24 h. The cells were treated with IC_50_ and IC_70_ concentration of CoCl_2_·H_2_O for 12–14 h. Thereafter, the cells were washed with phosphate buffer saline and incubated with 4 % PFA for half an hour. After fixation, the cells were stained with 4,6-diaminidino-2-phenylindole (DAPI), and slides were prepared for imaging with confocal microscope. The slides were scanned under Nikon eclipse TiE inverted fluorescence microscope equipped with a Nikon AiR laser scanning confocal microscope system (Nikon Corporation, Japan). Fluorescence was observed with long pass 488 emission filters.

### Scanning electron microscopy

For scanning electron microscopy, the formerly described method was followed (Ye et al. 2012). The PC-3 cells at concentration of 5 × 10^5^ cells/well in 6 well plate were seeded. After 24 h, the cells were treated with IC_50_ concentration of CoCl_2_·6H_2_O for 12–14 h. Thereafter, the cells were fixed in 4 % osmium tetraoxide. The cells were dehydrated using concentration gradient of ethanol and acetone before freeze drying under vacuum in lyophilizer. The cells were then gold plated using Quarum Q150R ES Rotary-pumped sputter coater. Finally, the cells were observed and photographed under EVO LS 10 scanning electron microscope (Carl Zeiss, Germany).

### Cell cycle analysis

Cell cycle analysis was done on PC-3 cells by previously described method (Carnevale et al. 2014). The PC-3 cells at a concentration of 5 × 10^5^ were grown in 6-well plate overnight, and afterwards the cells were treated with different concentrations of CoCl_2_·6H_2_O for 24 h. After treatment, the cells were trypsinized and centrifuged at 1500 rpm for 5 min and pellet was washed thrice with PBS. Thereafter, cells were fixed in 1 mL ice cold 70 % ethanol for 3 h at −20 °C. Cells were incubated with DNase free RNase at 37 °C for 1 h and then stained with propidium iodide (5 µg/mL) for 20 min at 4 °C in dark. These fixed and stained cells were then analysed immediately on BD Accuri C6 flow cytometer to study the different phases of cell cycle.

### LDH assay

The LDH assay was performed by the method of Linford and Dorsa 2002 with slight modifications. The 96 well plate was seeded with PC-3 cells at density of 1 × 10^4^ cells/well and allowed to adhere for 24 h. Thereafter, the cells were treated with IC_50_ and IC_70_ concentrations of CoCl_2_·6H_2_O for 12–14 h. After treatment, 50 µL of the culture supernatant was transferred to new 96-well culture plates and mixed with 50 µL of the LDH substrate mixture. The reaction was stopped by adding 100 µL of solution containing 50 % dimethylformamide and 20 % sodium dodecyl sulfate (DMF/SDS, pH 4.7). Absorbance was measured at 570 nm with Biotek synergy HT ELISA reader (Thermo Scientific).

### Annexin V-FITC/PI assay

Apoptosis induced by treatments of CoCl_2_·6H_2_O in PC-3 cells was analysed using Annexin V-FITC detection kit (Sigma Aldrich Inc., USA.). The procedure was followed as given in the kit. The cells were viewed under Nikon eclipse TiE inverted fluorescence microscope equipped with a Nikon AiR laser scanning confocal microscope system (Nikon Corporation, Japan).

### ROS generation

The PC-3 cells were used to examine the ROS generation by method of Shin et al. 2009 with slight modifications (Lippi et al. 2005). The PC cells at concentration of 5 × 10^5^ were seeded in 12 well plate. After 24 h, the cells were treated with IC_50_ and IC_70_ concentration of CoCl_2_·6H_2_O for 12–14 h. The treated cells were stained with 10 µg/mL of 5,6-chloromethyl-2′-7′dichlorodihydroflurescein diacetate (H_2_DCFDA). Then, fluorescence was measured using ELISA plate reader (Bio Tek Multi Mode Reader) with excitation and emission wavelengths of 488/20 and 530/20 nm respectively.

### Measurement of MMP

The changes in mitochondrial membrane potential in treated PC-3 cells were assessed using the method (Deng et al. 2013). The overnight grown PC-3 (5 × 10^5^) cells were treated with IC_50_ and IC_70_ concentrations of CoCl_2_·6H_2_O for 12–14 h and followed by staining with rhodamine 123 fluorescence dye (1 µM) for 45 min. Cells were washed thrice with PBS (1×). Thereafter, the rhodamine 123 fluorescence was examined using ELISA plate reader (Bio Tek Multi Mode Reader) with excitation of 488 nm and emission of 530 nm.

### Measurement of caspase activity

The caspase activity was measured in PC-3 cells treated with IC_50_ and IC_70_ concentrations of CoCl_2_·6H_2_O for 12–14 h using the method described in caspase kit purchased from Biovision Inc., USA.

### Statistical analysis

The experimental data was analysed in MS-Excel using self-coded software. Two-way analysis of variance (ANOVA) was done to check the significance of differences between and within treatments, and interactions if any. The null hypothesis tested was that there is no significant difference among the means. The alternative hypothesis was at least two means differ from each other. HSD was calculated using Tukey’s multiple comparison test. Chi square test was used to check whether there is a significant difference between the expected frequencies (positive control) and the observed frequencies (different biochemical parameters like LDH, ROS, MMP and caspase). Significance levels of F-ratios and chi- square were checked at P < 0.001. Logarithmic regression analysis was done to determine the significance of correlativity among the variables (Sokal and Rohlf 1981; Bailey 1994). Camptothecin (10 µM) was used as positive standard.

## Results

### CoCl_2_·6H_2_O induces cell death in cancer cell

Three forms of Cobalt i.e., CoCl_2_·6H_2_O, Macro-Co(II,III) oxide and Nano-Co(II,III) oxide were tested for antiproliferative activity against PC-3 cancer cell line as shown in Table 1. Among these, CoCl_2_·6H_2_O showed maximum antiproliferative activity of 79.56 % at highest tested dose of 100 mg/L followed by 13.70 and 14.12 % of Macro-Co(II,III) oxide and Nano-Co(II,III) oxide respectively. Since, CoCl_2_·6H_2_O was found to the most effective antiproliferative agent hence it was further studied against IMR3, A549 and 293T cell lines as well. CoCl_2_·6H_2_O inhibited rapid proliferation of cancer cells and induced maximum cell death in IMR-32 followed by PC-3 and A549 with IC_50_ values of 7.12, 21.91 and 29.81 mg/L respectively. CoCl_2_·6H_2_O was found to be more toxic to cancer cell lines as compared to the normal cell line (Table 2).

**Table 1:** Percent inhibition of prostrate (PC-3) cancer cell lines treated with different concentration of CoCl_2_·6H_2_O, Macro-Co(II,III) oxide and Nano-Co(II,III) oxide

Conc. (mg/L)	CoCl_2_·6H_2_O	Macro-Co(II,III) oxide	Nano-Co(II,III) oxide
	% Inhibition		
10	33.13	3.94	−12.31
25	46.86	5.95	−4.47
50	61.45	8.81	6.29
75	71.12	10.56	9.93
100	79.56	13.70	14.12

**Table 2:** Percent inhibition, IC_50_ values of various cell lines treated with different concentrations of CoCl_2_·6H_2_O (5–100 mg/L) by using best fit regression model

Concentration of CoCl_2_·6H_2_O (mg/L) (x)								
Cell line	Inhibition (%) (y)						Regression equation	IC_50_
	5	10	25	50	75	100		
293 T	7.27	16.97	27.73	47.26	62.67	69.72	y = 20.92 ln(x) − 31.03 R = 0.977***	48.1
IMR-32	31.24	66.60	77.22	78.10	79.01	79.22	y = 13.59 ln(x) + 23.33 R = 0.849***	7.12
PC-3	19.80	31.58	49.70	67.98	80.36	82.08	y = 21.86 ln(x) − 17.49 R = 0.995***	21.91
A549	0.817	5.48	46.92	73.61	80.02	80.79	y = 30.61 ln(x) − 53.93 R = 0.980***	29.81

Two-way ANOVA summary table						
Source of variation	df	SS	MSS	F-ratio	Tukey’s HSD_0.05_	4.79
Treatment (cell line)	3	8632.36	2877.45	470.06***		
Dose (CoCl_2_·6H_2_O)	5	39,715.85	7943.17	1297.59***		
Treatment × dose (interaction)	15	5669.54	377.97	61.75***		
Error	48	293.83	6.12			
Total	71	54,311.57				

R *** P < 0.001; *** P < 0.001; n = 5

### Non revival of treated PC-3 and 293T cells

**Fig. 1:**
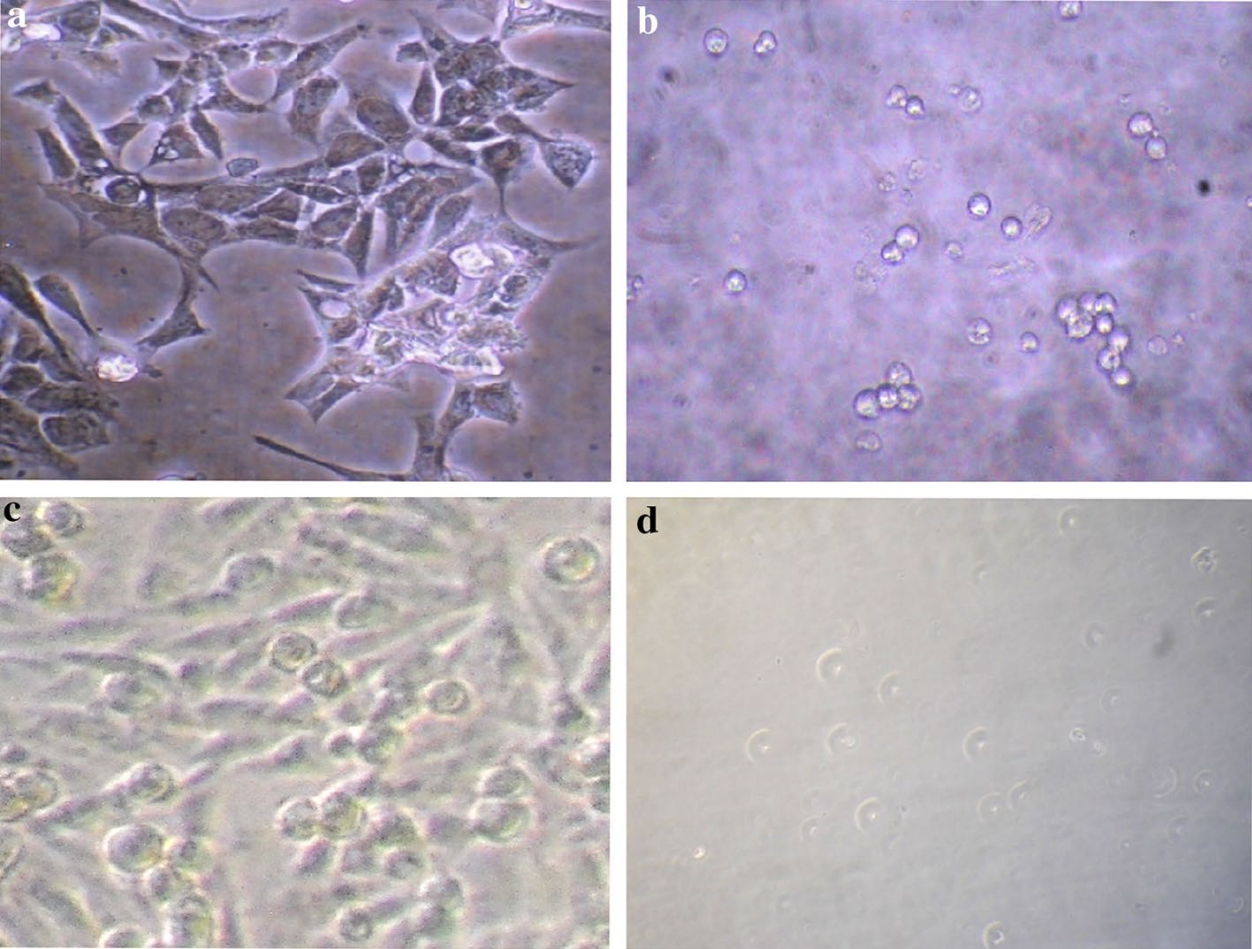
Subculturing of dead 293T and PC-3 cells after treatment with CoCl_2_·6H_2_O after 24 h. **a** 293T cells without treatment, **b** 293T cells with CoCl_2_·6H_2_O (IC_50_) treatment, **c** PC-3 cells without treatment, **d** PC-3 cells with CoCl_2_·6H_2_O (IC_50_) treatment

Figure 1 shows harvested 293T and PC-3 cells with and without treatment with CoCl_2_·6H_2_O. It was observed that treated PC-3 as well as 293T cells did not revert back to normalcy even after transferring to fresh growth medium.

### CoCl_2_·6H_2_O induces morphological and chromatin changes

**Fig. 2:**
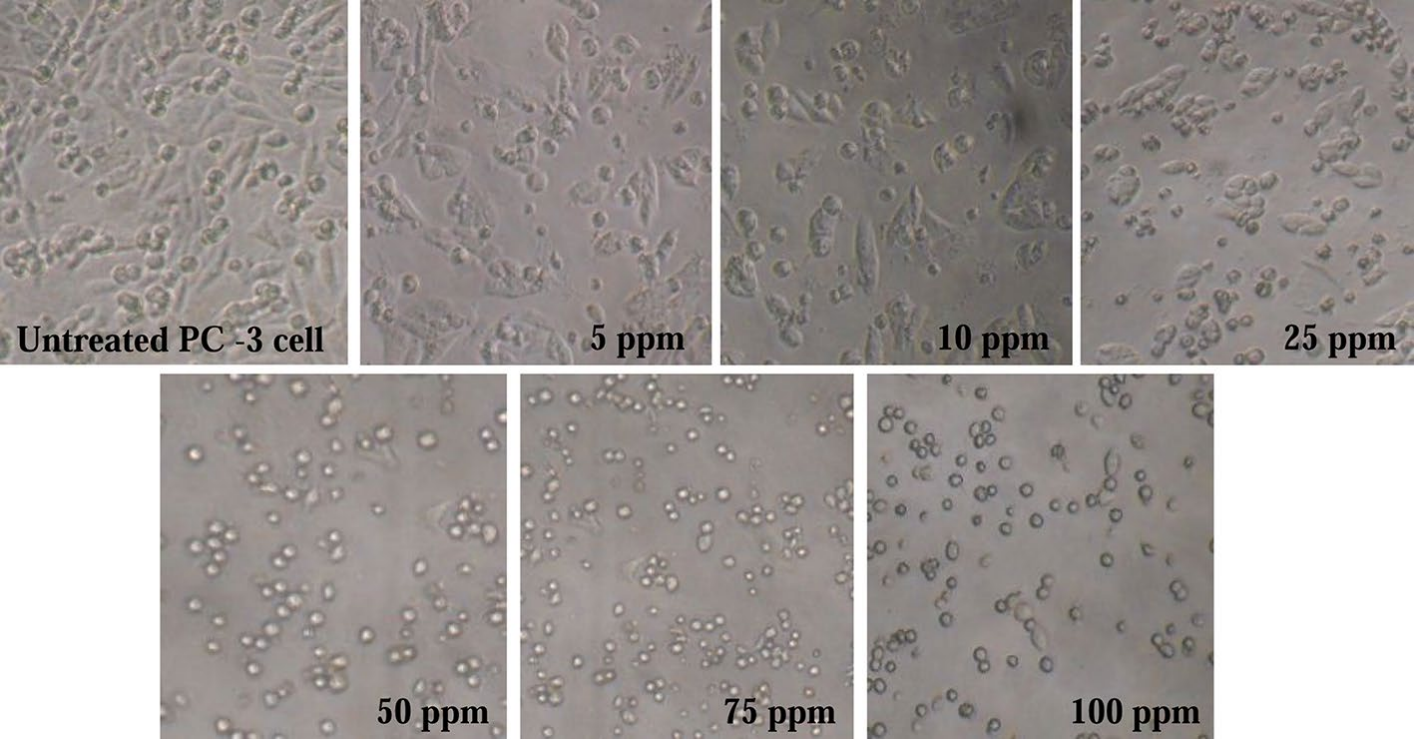
Phase contrast micrographs (40X, Nikon eclipse TS100) of CoCl_2_·6H_2_O concentrations effect (5–100 mg/L) on morphology of PC-3 cells observed after 12–14 h of treatment at magnification of ×40

**Fig. 3:**
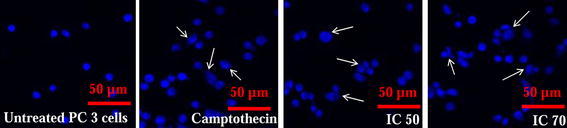
Confocal micrographs (Nikon eclipse TiE inverted fluorescence microscope, Nikon Corp. Japan) of effect of CoCl_2_·6H_2_O  treatment (IC_50_ and IC_70_, 12–14 h treatment) on morphology of PC-3 cells

**Fig. 4:**
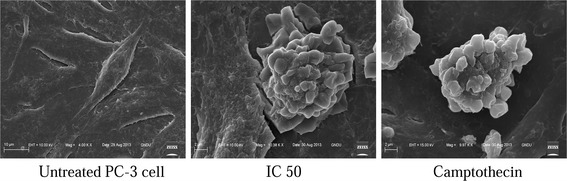
Scanning electron micrographs (EVO LS 10. Carl Zeiss Germany) of effect of CoCl_2_·6H_2_O (IC_50_, 12–14 h treatment) on morphology of PC-3 cells

The microscopic analysis of PC-3 cells treated with various concentrations of CoCl_2_·6H_2_O showed the details of the morphological and chromatin changes. Light microscopy showed that with increasing concentration of CoCl_2_·6H_2_O, the cell morphology was changed involving cell rounding (Fig. 2). Confocal microscopic studies revealed the nuclear morphological changes induced by CoCl_2_·6H_2_O at chromatin level. Changes like chromatin breaks and chromatin condensation were observed in CoCl_2_·6H_2_O treated PC-3 cells (Fig. 3). Further, scanning electron microscopy revealed the appearance of apoptotic bodies in PC-3 cells treated with CoCl_2_·6H_2_O (Fig. 4).

### CoCl_2_·6H_2_O arrests PC-3 cells ate sub go phase of cell cycle

**Fig. 5:**
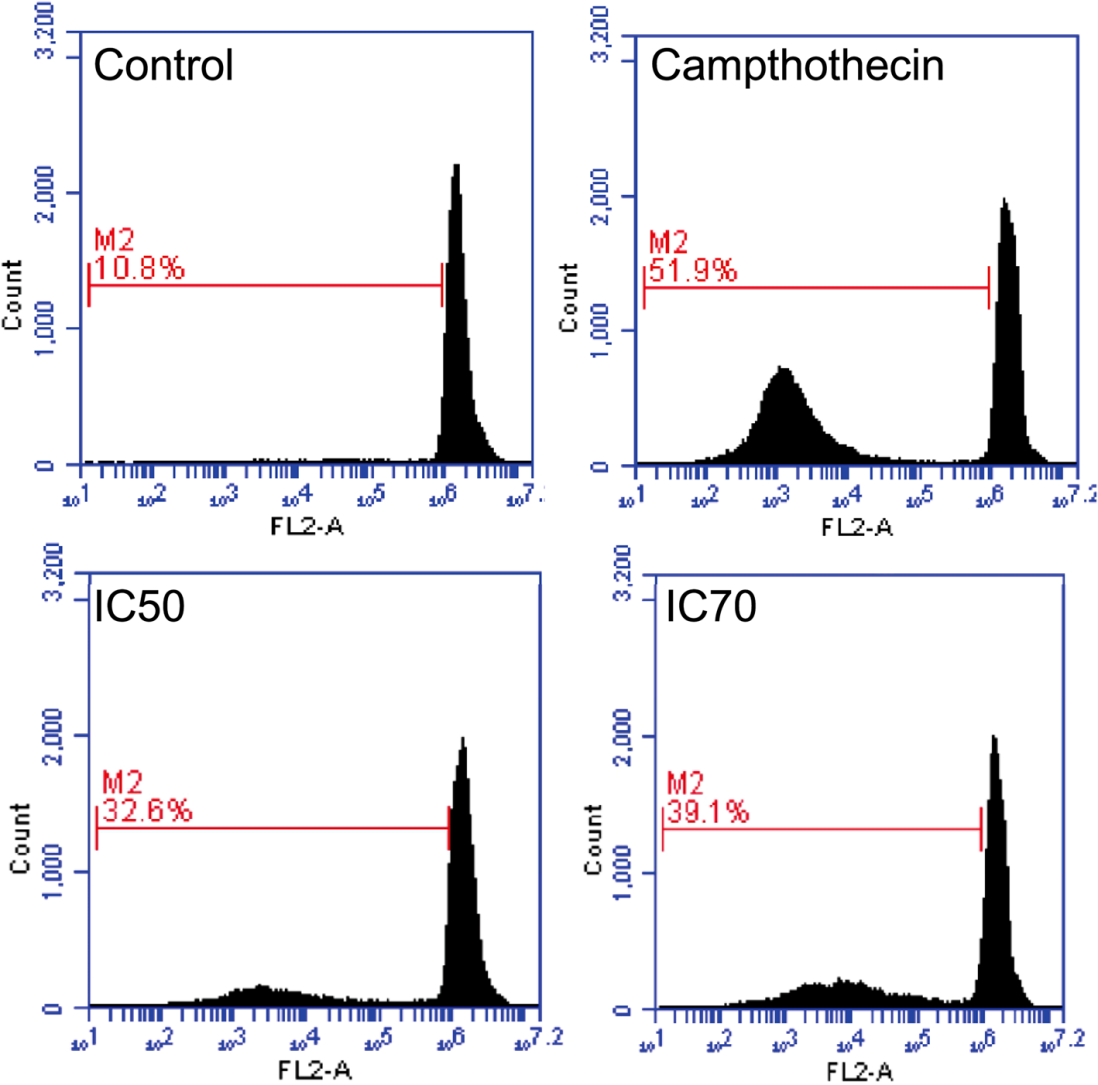
Cell cycle analysis in PC-3 cells of untreated control, positive control (campthothecin), IC_50_ and IC_70_ treatment of CoCl_2_·6H_2_O. Cells were stained with PI (5 µg/mL for 20 min) and analysed using BD Accuri C6 flow cytometer

The cell cycle arrest induced in PC-3 cells treated with IC_50_ and IC_70_ concentrations of CoCl_2_·6H_2_O is shown in Fig. 5. The untreated PC-3 showed only 10.8 % of cells in sub G_o_ phase of cell cycle while this number increased to 32.6 and 39.1 % with treatment of IC_50_ and IC_70_ concentrations of CoCl_2_·6H_2_O respectively. The positive control, camptothecin treatment showed 51.9 % of cells in sub G_0_ phase.

### CoCl_2_·6H_2_O induces LDH release

The LDH assay was employed to study the effect of CoCl_2_·6H_2_O on integrity of PC-3 cell membrane. Both the IC_50_ and IC_70_ treatments of CoCl_2_·6H_2_O induced release of 23.2 and 38.2 % of LDH respectively. The positive control camptothecin resulted in 26.7 % release of LDH over the untreated PC-3 cells (Table 3).

**Table 3:** Change in LDH, ROS, MMP and caspase activities at IC_50_ and IC_70_ concentrations of CoCl_2_·6H_2_O for 12–14 h treatment in PC-3 cancer cell line with respect to untreated PC-3 cells

	LDH	ROS	MMP	Caspase
Untreated PC-3 cells	100	100	100	100
Camptothecin (10 µM) treated PC-3 cells	126.7	176.5	72.7	174.1
CoCl_2_·6H_2_O (IC_50_) treated PC-3 cells	123.2	152.9	80	170.4
CoCl_2_·6H_2_O (IC_70_) treated PC-3 cells	138.2	211.8	73.3	177.8
χ^2^	27.07***	211.42***	18.55***	164.88***

IC_50_ and IC_70_ doses calculated using regression equation: y = 21.86 ln(x) − 17.49

*** P < 0.001; n = 3

### CoCl_2_·6H_2_O induces apoptosis

**Fig. 6:**
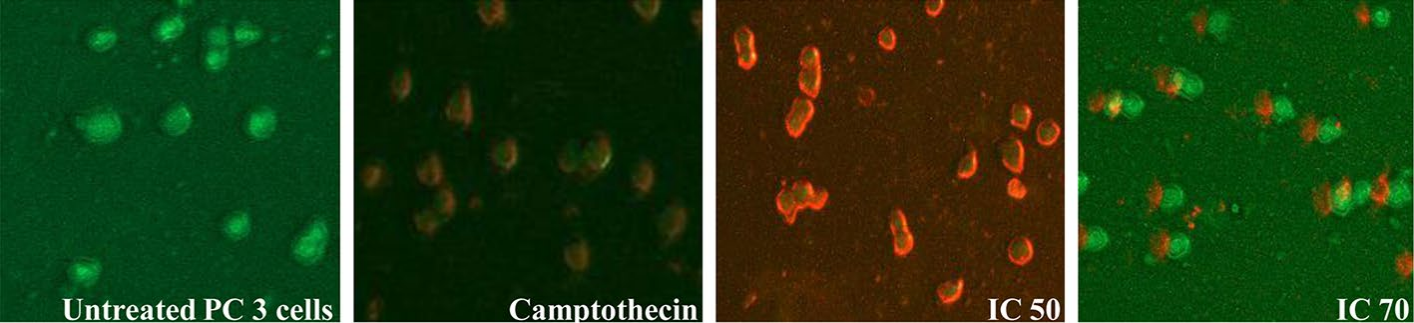
Annexin V/propidium iodide staining for confirmation of apoptosis and necrosis in PC-3 cells with IC_50_ and IC_70_ treatments of CoCl_2_·6H_2_O in comparison to control and camptothecin (10 µM) as positive standard

In order to confirm the apoptotic or necrotic mode of cell death in PC-3 cells, Annexin V/propidium iodide staining dyes were used. Fluorescently labelled annexin gets strongly bound to phosphatidylserine part in plasma membrane of cells upon onset of apoptosis, while propidium iodide was used for conferring necrosis. The cells showed mild necrosis but peculiar features of apoptosis (Fig. 6).

### CoCl_2_·6H_2_O induces ROS generation

The generation of ROS in PC-3 cells treated with IC_50_ and IC_70_ concentrations of CoCl_2_·6H_2_O was measured spectrofluorimetrically. The results were presented as relative fluorescence as compared to the untreated cells. The CoCl_2_·6H_2_O treatments showed dose dependent increase in ROS generation. More than two folds of increase in ROS generation was observed in PC-3 cells treated with IC_70_ concentration of CoCl_2_·6H_2_O while positive standard camptothecin induced 1.76-fold increase (Table 3).

### CoCl_2_·6H_2_O disrupts mitochondrial membrane potential

The disruption of mitochondrial membrane is a key step in the induction of apoptosis in cancerous cells. The results were presented as percentage rhodamine intensity. The intensity of rhodamine detected in untreated cells was considered as 100 %. The treatment with IC_50_ and IC_70_ concentrations of CoCl_2_·6H2O showed dose dependent decrease in rhodamine intensity as detected by spectrofluorimeter. Positive standard camptothecin showed 72.7 % while IC_50_ and IC_70_ showed up to 80 and 73.3 % of rhodamine intensity (Table 3).

### CoCl_2_·6H_2_O activates caspase 3

Caspase-3 activity was assessed in PC-3 cells treated with IC_50_ and IC_70_ concentrations of CoCl_2_·6H_2_O (Table 3). A dose dependent increase in caspase activity was observed in PC-3 cells treated with CoCl_2_·6H_2_O. The IC_50_ and IC_70_ concentrations of CoCl_2_·6H_2_O showed significant increase in caspase activity of 170.4 and 177.8 % respectively as compared to untreated PC-3 cells. The positive control camptothecin showed 174.1 % increase in caspase activity as compared to the untreated PC-3 cells.

## Discussion

Metals and their complexes have been extensively used for their remedial properties since ages. Recent chaos over their toxic potentials has overlooked their medicinal values, thus warranting a need to delineate the ailment preventive effects of metals substantiated with a scientific data. Recently the mechanism of action of metal ions in cancer cells has been elucidated. It has been reported that metal ions induce hypoxia in cellular entity and resulting in activation of various signalling pathways and hypoxia inducible transcription factors (HIF) (Salnikow et al. 2000; Huang et al. 2013). Cobalt chloride is well known for inducing hypoxia like conditions both in vivo (Alexa et al. 2015) and in vitro (Shweta et al. 2015) conditions. Medicinally, it is also used to treat anaemic patients. Having known its medical uses, it would be pertinent to test its anti-proliferative and anti-cancer activities.

Preliminary, three forms of cobalt were screened for antiproliferative active against PC-3 cancer cell line using MTT assay. MTT is the most widely widely used method to investigate antiproliferative potential of test compound against various cell lines (Dellai et al. 2012; Pieme et al. 2013; Rapisarda et al. 2015). The mechanism behind this assay includes conversion of yellow coloured MTT into blue colored formazon by the action of the enzyme succinate dehydrogenase within mitochondria of living cells. Our results showed CoCl_2_·6H_2_O as the most active antiproliferative agent, hence it was further tested against other cancer cell lines. Cell death induced by CoCl_2_·6H_2_O inhibited the formation of blue colored formazan crystals. Lower the intensity of blue colour, more will be the antiproliferative potential of the test compound. CoCl_2_·6H_2_O was tested at 6 concentrations ranging from 5 to 100 mg/L against four cell lines viz. 293T, PC-3, A549 and IMR-32. It induced maximum cell death in IMR-32 cancer cell line. Similar studies were conducted by (Martinez-Built et al. 2015; Villa-Pérez et al. 2016) using Co(II), Ni(II), Zn(II) and Cu(II) complexes against HeLa, HTC-15, MCF-7 and PC-3 cell lines and they established that ligand binding capability of transition metals is responsible for their antiproliferative activity. Although, the growth of normal non cancerous cells was also inhibited to certain degree but interestingly, this growth inhibition was more in case of cancer cell lines. Further, the LDH assay was also performed to check whether the mode of cell death induced by test compound is apoptosis or necrosis. The results clearly showed a very mild release of LDH from cell culture treated with CoCl_2_·6H_2_O. This prompted us to look for other way of cell death i.e., apoptosis. Further studies were conducted on PC-3 cells to eke out the apoptotic signatures from both inside and outside of the cell. All the microscopic studies on CoCl_2_·6H_2_O treated PC-3 cells showed the appearance of apoptotic bodies, blebbing, condensation of chromatin and shrinkage of cells. These morphological and cytological changes in PC-3 cells point towards the apoptotic mode of cell death (Brauchle et al. 2014). Similar apoptotic inducing activities of cobalt were studied by Akita et al. (2007) where they found that salts of cobalt reduces expression of Bcl-2 protein at transcriptional as well as translational level in HSG cells and induces apoptosis. The cell cycle analysis using flow cytometry showed an initial PC-3 cell arrest at sub G_o_ phase of cycle. Most of the anticancer drugs act by first arresting the cancer cells at particular phase of cell cycle and then start inducing apoptosis (Pathania et al. 2015). Similar studies were conducted by Van Rijt et al. (2014) and they observed cell arrest in S-phase after treatment with organometallic osmium compounds in A549 cancer cells. The cell surface markers change their positions during apoptosis and to confirm this annexin V-FITC/propidium iodide staining was done and images of stained cells were taken by confocal microscope. The pattern of staining showed the DNA damage and movement of apoptotic markers to cell surface to which annexin was bound. It was further tested that whether apoptosis was operating by intrinsic or extrinsic pathway. The treated and untreated PC-3 cells were checked spectroflurimetrically for ROS and MMP using different dyes. Results showed that CoCl_2_·6H_2_O induced generation of ROS which in turn damaged mitochondria as was evident from MMP reading. These results were in accordance with studies by Zhang et al. (2016) where Co(II) complexes were found to alleviate ROS and disrupted mitochondrial membrane in cancer cell lines. The intrinsic pathway requires an induction like overproduction of ROS, radiations that damage mitochondria which in turn activate caspases to bring out apoptotic death (Hajrezaie et al. 2015; Elmore 2007; Tian et al. 2015; Leverson et al. 2015). Thus, caspases being an important class of cysteine proteases play requisite role in apoptosis (Exile et al. 2014). Our results showed a dose dependent increase in caspase activity in PC3 cells with an increase in the concentration of CoCl_2_·6H_2_O. Similar results were observed in ruthenium polypyridyl complexes, which lead to increased caspase-3 activity in breast cancer cells (Cao et al. 2015).

Thus, it could be inferred that the hypoxic conditions induced by CoCl_2_·6H_2_O deprives the proliferating cancer cells of the oxygen required for their metabolism. This could have increased the expression of HIF transcription factor and the over expression of later is an inducer of apoptosis (Krick et al. 2005). Thus apoptosis was found to execute in PC3 cells by over generation of ROS and concomitant damage to mitochondrial membrane.

## Conclusions

The present work unequivocally demonstrates the antiproliferative potential of CoCl_2_·6H_2_O against brain (IMR-32), lung (A549) and prostrates (PC-3) cancer cell lines. CoCl_2_·6H_2_O increased ROS production, decreased mitochondrial membrane potential, increased the activity of casapse-3 and sub G_0_ population of cells. The microscopic studies further confirmed the cellular and nuclear morphological changes following CoCl_2_·6H_2_O treatment. The mechanism of cellular death in PC-3 is apoptosis. Further, in vivo studies are required to investigate the anticancerous properties of cobaltous chloride hexahydrate.
